# Growth differentiation factor-15 may be a novel biomarker in pancreatic cancer: A review

**DOI:** 10.1097/MD.0000000000036594

**Published:** 2024-02-09

**Authors:** Meng Guo, Hui Zhao

**Affiliations:** a Tongren Hospital, Shanghai Jiao Tong University School of Medicine, Shanghai, China.

**Keywords:** CA199, GDF-15, MIC-1, pancreatic cancer, tumor marker

## Abstract

Pancreatic cancer is a highly malignant and invasive gastrointestinal tumor that is often diagnosed at an advanced stage with a poor prognosis and high mortality. Currently, carbohydrate antigen199(CA199) is the only biomarker approved by the FDA for the diagnosis of pancreatic cancer, but it has great limitations. Growth differentiation factor-15 (GDF-15) is expected to be a novel biomarker for the diagnosis, efficacy prediction, and prognosis assessment of pancreatic cancer patients. In this paper, we searched the keywords GDF-15, macrophage inhibitory cytokine-1 (MIC-1), CA199, pancreatic cancer, and tumor markers in PubMed and Web of Science, searched related articles, and read and analyzed the retrieved papers. Finally, we systematically described the characteristics, mechanism of action, and clinical value of GDF-15, aiming to provide help for the detection and treatment of pancreatic cancer.

## 1. Introduction

Due to the lack of typical symptoms and signs in the early stages, as well as its aggressive nature and high malignancy, pancreatic cancer often has a grim prognosis and high mortality rate. It is projected to become the second leading cause of cancer-related deaths by 2030.^[1,2]^ Surgical resection is the most effective method for treating patients with pancreatic cancer at present. However, only a small percentage (15%–20%) of newly diagnosed patients are eligible for surgery. In comparison, the majority (80%) are diagnosed with either locally advanced or metastatic pancreatic cancer, rendering them ineligible for surgery. These patients often have to resort to palliative treatments such as chemotherapy, radiotherapy, and immunotherapy. Unfortunately, the results and prognosis of these treatments are not favorable. Therefore, early detection and treatment are crucial for improving outcomes in pancreatic cancer patients.^[3,4]^ Due to its universality and rapid imaging characteristics,CT is the first choice for evaluating patients with suspicious conditions. However, CT has a low detection rate for isodense lesions and small masses. On the other hand, MRI has better resolution compared to CT. Nevertheless, MRI has some disadvantages, such as the absence of standardized parameters for advanced imaging sequences, limited spatial resolution, susceptibility to patient movement and intestinal gas, and challenges for patients with claustrophobia.^[5,6]^ Endoscopic ultrasound has better sensitivity and specificity for detecting pancreatic cancer, and it is more accurate in detecting small lesions (0.5–2 cm).^[7]^ However, experiments conducted on animal models have shown that changes in molecular markers occur before structural changes are detected by imaging techniques in the pancreas. As a result, more studies have begun to focus on identifying molecular markers.^[8]^

Tumor markers are produced and secreted by tumor tissues. A good tumor marker should have high specificity and sensitivity, good predictability, and correlate with the tumor stage. However, it is important to note that no tumor marker possesses 100% sensitivity and 100% specificity.^[9]^ To date, the glycoantigen CA199 is the only biomarker approved by the FDA to diagnose pancreatic cancer. However, it still has many shortcomings in diagnosing pancreatic cancer, including low average sensitivity and specificity for CA199 and a positive predictive value of only 0.5% to 0.9%. Patients who lack the Lewis antigen may have false-negative CA199 results. Additionally, CA199 levels can also be elevated in cirrhosis, pancreatitis, benign pancreaticobiliary obstructive disease, and especially biliary disease.^[10–12]^ Despite multiple studies indicating a significant increase in overall survival with lower CA199 serum levels (20%–89%), a recent large-scale investigation by Hess et al did not support these findings. The use of CA199 to assess the prognosis of pancreatic cancer patients after first-line palliative treatment remains a topic of debate.^[9]^

### 1.1. Features of the GDF-15

GDF-15, also known as MIC-1, Placental Bone Forming Protein (PBFP), Placental Transforming Growth Factor (PTGF), and Non-steroidal Anti-Inflammatory Drug Activating Aene-1 (NAG-1), is typically not detectable under normal physiological conditions except in the placenta and some neural tissues. It is a member of the transforming growth factor beta superfamily, originally found in macrophages.^[13–15]^ In contrast, GDF-15 is substantially expressed in tumors and the tumor microenvironment; elevated levels of GDF-15 are directly associated with the emergence and spread of malignancies.^[16,17]^ The GDF-15 gene in humans is located on chromosome 19 at position p12-13.2. The entire gene is composed of approximately 7007 base pairs, with the coding region consisting of 2746 base pairs. This coding region has 2 exons, separated by an intron about 1820 base pairs long. The human GDF-15 preprotein contains 308 amino acids, including a signal peptide consisting of 29 amino acids at the n-terminus, a 167-amino-acid prepeptide, and a mature region consisting of 112 amino acids in the C-segment, and the n-terminal hydrophobic signal peptide sequence of the GDF-15 precursor is removed during protein hydrolysis.^[18,19]^ Mature GDF-15 has 7 cysteine residues, 6 of which form a cysteine structural domain, a key structural feature of members of the TGF-β superfamily. Two structural domains are linked by a disulfide bond to form a homodimer, which is sheared by a furin-like protease at the amino acid target sequence RXXR to form secreted GDF-15. Secreted GDF-15 binds to the extracellular matrix and cleaves to active proteins in response to environmental stimuli such as inflammation.^[19–22]^ GDF-15 has a unique secretory pathway. It is synthesized in the cytoplasm, transported to the nucleus, and released into the extracellular matrix upon reentering the cytoplasm. The active form of GDF-15 is a dimer that is released during maturation. Considering the various types of biosynthesis and their interactions, the mature dimer, intracellular form, and propeptide of GDF-15 may all play crucial roles in regulating its biological effects.^[23]^

### 1.2. GDF-15 and epigenetics

GDF-15 expression is epigenetically regulated in several tumor cell lines. Clonal analysis revealed that the GDF-15 promoter upstream consists of binding sites for a variety of essential transcription factors, including specificity protein-1 (Sp1), early growth response protein-1 (EGR-1), p53, and COUP transcription factor-1 (COUP-TF1).^[21,24]^ Wild-type p53 is a crucial tumor suppressor that exerts anti-tumor functions by causing cell cycle arrest and apoptosis in the downstream signaling pathways of DNA damage or cellular stress. It can activate the p53 binding site on the GDF-15 gene promoter, inducing the expression of GDF-15 to exert anti-tumor effects.^[15,25,26]^ As mentioned previously, in addition to p53, the activation of oncogenes EGR-1,GSK-3β, an C/EBP β can increase the expression of GDF-15.^[13]^ The study conducted by Husaini Y et al examined the impact of MIC-1/GDF-15 overexpression on the initiation and progression of prostate cancer. They found that although the tumors in these mouse models grew slower and had a lower tumor grade, they were more likely to metastasize. MIC-1/GDF-15, similar to TGF-β, has a complex effect on tumor behavior. It may suppress local growth while promoting metastatic spread.^[27]^ High concentrations of GDF-15 may exert pleiotropic functions at different stages of cancer.^[22]^

### 1.3. GDF-15 and the tumor microenvironment

The tumor microenvironment consists of immune cells, as well as other non-cancerous cells such as adipocytes, fibroblasts, blood vessels, signaling molecules, and the extracellular matrix.^[28]^ GDF-15 may promote immune evasion and immune rejection of tumor cells by modifying the tumor microenvironment, thereby facilitating tumor progression.^[17]^ There are 2 types of innate immune cells called macrophages: M1 and M2. The M2 phenotype is tumor-associated macrophages (TAMs), abundant in many tumors and contributing to tumor progression by promoting tumor growth and angiogenesis. On the other hand, the M1 phenotype represents the classical macrophage associated with acute inflammatory conditions and T-cell immunity. Its main function is to eliminate and destroy target cells.^[29]^ Early in tumorigenesis, macrophages play a significant role as the primary immune cell population. Activated macrophages can secrete cytokines, such as TNF-α and NO, or act as antigen-presenting cells to facilitate the destruction of tumor cells by activating cytotoxic T cells. NF-kB, a transcription factor, plays a crucial role in communicating between tumor cells and macrophages. Its activation promotes the proliferation, growth, and metastasis of cancer cells. In the early stages of tumorigenesis, the NF-kB/GDF-15 regulatory axis plays an important role in evading immune surveillance by macrophages. Elevated GDF-15 promotes NF-kB activation, inhibits macrophage activity, and blocks the synthesis of tumor necrosis factor TNF-α and NO through inhibition of TGF-β-activated kinase1 (TAK1), which leads to the immune escape of tumor cells.^[30]^ Fibroblasts in the tumor microenvironment play a crucial role in the development of pancreatic cancer. Tumor-infiltrating fibroblasts can acquire a persistently activated phenotype, leading to the excessive production of extracellular matrix proteins. These proteins accumulate and form fibrous connective tissues. As cancer cells proliferate uncontrollably, the growth of connective tissues creates pressure within the tumor, known as solid stress. This stress induces fibroblasts to secrete GDF-15. The upregulation of GDF-15 could facilitate the migration of pancreatic cancer cells in vitro.^[31,32]^Subsequent in vitro studies have shown that solid stress transcriptionally regulates GDF-15 expression by activating the AKT/CREB-1 pathway. Specifically, solid stress activates the Akt pathway, which phosphorylates CREB-1 and triggers its activation; activated CREB-1 functions as a transcription factor that directly binds to the promoter region of GDF-15. This binding leads to the secretion and self-secretion of GDF-15, promoting the migration of pancreatic cancer cells.^[33]^ NK cells play a significant role in anti-tumor activity. In vitro experiments have shown that GDF-15 reduces the lytic activity of NK cells, suggesting that GDF-15 causes the immunological escape of glioma cells by altering the function of NK cells.^[34]^ Dendritic cells (DCs) are professional antigen-presenting cells. It processes intracellular and extracellular antigens and produces peptides that bind to the major histocompatibility complex (MHC) to activate antigen-specific T cells for an immune response.^[35]^ Studies have shown that GDF-15 may cause the immunological escape of tumor cells by inhibiting the maturation of dendritic cells and impairing their antigen-processing function.^[36]^ The previous study demonstrated that overexpression of GDF-15 not only enhances the production of regulatory T-cells but also leads to the failure of DC cells by upregulating the immunosuppressive gene IDO.^[37]^ Considering that senescent cells can promote tumor progression by releasing a large number of chemokines, cytokines, metalloproteinases, and growth factors, and GDF-15 is involved in regulating the oncogenes P16 and P53 and inducing senescence, it is possible that GDF-15 may also contribute to tumor progression by inducing senescence in various cells within the tumor microenvironment.^[31,38]^ GDF-15 can influence immunological checkpoints, such as PD-1/PD-L1, to contribute to tumor development.^[17]^

### 1.4. Molecular mechanisms of GDF-15 action

Earlier studies have identified GDF-15 as a potential target for treating cachexia in patients with advanced cancer. Possible mechanisms involved in this treatment include hypothalamic TGF-βRII, ERK1/2, STAT-3, neuropeptide Y, and opioid nigocortin precursors.^[39,40]^ The mechanism became clearer with the discovery of glial-derived neurotrophic factor (GDNF) family receptor α-like (GFRAL), an extracellular protein encoded by exon 9 on human chromosome 6p12.1. GDF-15 has high affinity for GFRAL. The GFRAL-GDF15 complex binds to the tyrosine kinase co-receptor RET, which phosphorylates to initiate a signaling cascade and subsequently activates intracellular signaling pathways such as AKT, ERK1/2, and phospholipase C γ(PLC γ), but not the SMAD pathway.^[41,42]^ A recent study found that pancreatic cancer tissues have significantly higher levels of GFRAL expression compared to healthy pancreatic tissues. Stage IV pancreatic cancer tissues showed even higher levels of GFRAL expression than stage I. In addition, Kaplan–Meier analysis has shown that higher levels of GFRAL expression are associated with lower 5-year overall survival rates in patients. The study also found that GDF-15 promotes the growth and spread of pancreatic cancer cells through the GDF-15/GFRAL signaling pathway.^[43]^ GDF-15 can also act through other signaling pathways.NR5A2 is a transcription factor that regulates the expression of various oncogenes. Overexpressed NR5A2 directly binds to the promoter region of the GDF-15 gene and thus enhances the transcription of GDF-15 in pancreatic cancer and promotes the progression of pancreatic cancer.^[44]^ SULF2, an extracellular component whose high expression in pancreatic tissues or serum promotes pancreatic cancer growth and metastasis and correlates with a poor prognosis, was found to act through the activation of the TGFβ-SMAD2/3 pathway in pancreatic ductal carcinoma by GDF15.^[45,46]^ In the past few years, the mechanism of GDF-15 in other cancers has also been investigated. GDF-15 can bind to ErbB2, activate PI3K/AKT, and MAPK/ERK pathways, upregulate CyclinD1 and CyclinE1, and reduce p21, leading to the proliferation of cervical cancer cells.^[47]^ GDF-15 promotes cancer growth by inducing the activation of AKT, further phosphorylating GSK-3β, a downstream target of AKT in hepatocellular carcinoma, or further phosphorylating the ERK pathway in human breast cancer cells, gastric cancer cells, and multiple myeloma.^[48,49]^ In human hepatocellular carcinoma stem cell lines, GDF-15 may induce the growth and metastasis of stem cells by activating the AKT/GSK-3β-catenin pathway.^[26,50]^

### 1.5. GDF-15 in pancreatic cancer

GDF-15 is a valuable tumor marker for the diagnosis of pancreatic cancer.^[51]^ According to the findings of an earlier investigation by Koopmann et al, individuals with pancreatic cancer had higher serum levels of GDF-15 compared to those with benign pancreatic tumors, chronic pancreatitis, and healthy controls.^[52]^ Serum GDF-15 levels were found to be significantly higher in patients with pancreatic cancer compared to healthy individuals in a meta-analysis that involved 1235 pancreatic cancer patients and 730 healthy individuals.^[53]^ One study further showed that GDF-15 considerably outperformed CA199 in identifying healthy controls and patients with chronic pancreatitis from pancreatic cancer.^[2,54]^ For detecting pancreatic cancer in its earliest stages (stage IA), MIC-1/GDF-15 has the best diagnostic efficacy with a sensitivity of 62.5%, while CA199 has a sensitivity of 25.0%. Even in individuals with pancreatic cancer who tested CA199-negative, blood MIC-1 levels increased; enhanced MIC-1/GDF-15 levels were not related to serum CA199 levels.^55,56^A meta-analysis including 2826 subjects showed that although the sensitivity of MIC-1/GDF-15 was higher than that of CA199, the rate of missed diagnosis was lower than that of CA199. In terms of specificity, MIC-1/GDF-15 was slightly lower than CA199. This suggests that GDF-15 and CA199 play different roles in diagnosing pancreatic cancer.^[12]^ Serum MIC-1/GDF-15 can serve as a predictor of tumor formation in asymptomatic individuals at high risk of developing pancreatic cancer.^[55]^ In conclusion, GDF-15 is crucial for the detection of pancreatic cancer and may be more effective in certain patients, particularly those who do not express the Lewis antigen and have undetectable CA199.^[2,56]^

Numerous studies have shown that the combination of MIC-1 and CA199 significantly increases the sensitivity and specificity of diagnosing pancreatic cancer, as well as the ability to differentiate between pancreatic cancer and pancreatitis.^[8,55]^ Muhammad et al demonstrated that the combination of MIC-1/GDF-15 and alcohol dehydrogenase (ADH) increased the detection rate of pancreatic cancer.^[57]^ By combining miR-21 or miR-25 with MIC-1/GDF-15 and CA199, Yuan W. et al demonstrated that they could diagnose pancreatic cancer more accurately and distinguish it from other gastrointestinal tumors.^[3]^ Thus, combining GDF-15 with other tumor markers may improve the detection rate of pancreatic cancer. In addition, serum MIC-1/GDF-15 levels were associated with recurrence and prognosis of pancreatic cancer patients. Serum MIC-1 levels are significantly lower in patients with prostate cancer after surgical resection of the tumor, and they return to high levels upon recurrence.^[58,59]^ Patients survived on average 15.62 ± 2.44 months with MIC-1 levels ≥ 1932 ng/ml, compared to 18.66 ± 2.43 months in patients with low levels of MIC-1/GDF-15. This suggests that high levels of MIC-1/GDF-15 are associated with a poor prognosis.^[60]^

With significantly higher levels of MIC-1/GDF-15 in the pancreatic fluid of patients with pancreatic cancer who also had type 2 diabetes than in those who did not, as well as higher levels than type 2 diabetes combined with chronic pancreatitis, Kaur et al measured MIC levels through endoscopic collection of pancreatic fluid. This suggests a potential synergistic effect between type 2 diabetes and pancreatic cancer.^[61]^ It is crucial to minimize the influence of drugs as much as possible because multiple studies have demonstrated that NSAIDs impacts serum MIC-1/GDF-15 levels mainly through Egr-1 induction.^[21,62]^ Numerous additional clinical conditions, including chronic kidney disease, metabolic disorders, inflammation, and cardiovascular disease, may also lead to an increase in GDF-15 levels.^[21]^

Fluorouracil, platinum, and gemcitabine are commonly used chemotherapy drugs for treating pancreatic cancer. Twist, a member of the helix-loop-helix family of transcription factors, is upregulated in pancreatic cancer tissues and to contributes to the progression of pancreatic cancer. Twist overexpression boosted p38 MAPK activity, significantly increased GDF-15 levels, elevated cisplatin half-maximal inhibitory concentration (IC50) values, and resulted in cisplatin resistance in ASPC1 and BXPC3 human pancreatic cancer cells.^[63]^ In addition, an increase in GDF-15 levels in individuals receiving platinum-based chemotherapy can worsen side effects and diminish their quality of life.^[64]^ A monoclonal antibody (mAB1) that neutralizes circulating GDF-15 reduces cisplatin-induced vomiting and cachexia, potentially improving quality of life and survival.^[64]^ It has been demonstrated that GDF-15 can increase the chemosensitivity of pancreatic cancer cells to gemcitabine.^[43]^ GDF-15 interferes with T cell activation by dendritic cells and the infiltration of activated T cells into the tumor microenvironment by inhibiting dendritic cell maturation and blocking immune cell recruitment. Immune checkpoint inhibitors may not be effective in treating cancer if there is no tumor microenvironment where T lymphocytes have infiltrated. One example of a cold tumor with a limited treatment response is pancreatic cancer. It is vital to transform this “cold” tumor, which is rejected or ignored by immune cells, into a “hot” tumor that is well-infiltrated and responsive to immunotherapy. Using the GDF-15 antibody to neutralize GDF-15 could be beneficial for immunotherapy.^[17,65]^

## 2. Conclusion

As a tumor marker, GDF-15/MIC-1 has great potential for the diagnosis, staging, and prognosis of pancreatic cancer. The GFRAL receptor has been confirmed to be the sole receptor for GDF-15/MIC-1, but its role in pancreatic cancer and related mechanisms need to be confirmed by more of our experimental data. As mentioned above, pancreatic cancer has a significantly increased ability to secrete GDF-15, and high levels of GDF-15 are strongly associated with the poor prognosis and survival of pancreatic cancer patients. When combined with other available biomarkers, such as CA199 and ADH, MIC-1/GDF-15 can improve the sensitivity and specificity of pancreatic cancer diagnostics. Additionally, GDF-15 as a therapeutic target has significant clinical value in reducing anorexia and weight loss in tumor patients, reversing resistance to chemotherapy drugs, and preventing the spread of cancer. At present, many medications that target GDF15 are now undergoing clinical trials and are anticipated to go on sale shortly, giving sufferers new hope.

## Author contributions

**Conceptualization:** Meng Guo, Hui Zhao.

**Data curation:** Meng Guo.

**Validation:** Hui Zhao.

**Writing – original draft:** Meng Guo.

**Writing – review & editing:** Meng Guo, Hui Zhao.

## Correction

This article was originally published with affiliation a appearing as “Shanghai Jiaotong University School of Medicine affiliated Tongren Hospital, Shanghai, China.” It has now been updated to “Tongren Hospital, Shanghai Jiao Tong University School of Medicine, Shanghai, China.”
